# Double-chirped mirrors expand the bandwidth of infrared frequency combs

**DOI:** 10.1038/s41377-025-02078-4

**Published:** 2025-11-13

**Authors:** Jacob B. Khurgin

**Affiliations:** Department of ECE, Johns Hopkins University, Baltimore, MD USA

**Keywords:** Semiconductor lasers, Infrared spectroscopy

## Abstract

A novel dispersion-compensation scheme based on double-chirped Bragg mirrors is implemented in a mid-infrared quantum cascade laser. As a result, stable and broadband frequency combs are generated, which are indispensable for high-precision applications in spectroscopy and metrology.

Optical frequency combs (FCs)^1^ are coherent sources of electromagnetic radiation characterized by a spectrum of equally spaced, narrow spectral lines and a periodic temporal waveform. A defining feature of FCs is the mutual coherence of their spectral components—meaning that the phases of individual lines are locked relative to each other. Since their first experimental demonstration a few decades ago^2,3^, FCs have become indispensable tools in metrology, high-resolution spectroscopy, microwave photonics, and optical clocks.

Traditionally, FCs are generated either using mode-locked lasers^4^ or through nonlinear optical processes in high-Q resonators^5^. While frequency comb generation in the visible and near-infrared (NIR) regions is now well established, extending this technology into the mid-infrared (MIR) and, in particular, the long-wave infrared (LWIR, 8–13 μm) has posed significant challenges^6^. Historically, LWIR combs have been obtained indirectly by nonlinear frequency downconversion of visible or NIR combs.

A major breakthrough came with the discovery of frequency comb formation in free-running quantum cascade lasers (QCLs) operating in the Mid-IR^7^ and terahertz (THz)^8^ regimes. Remarkably, these QCL-based combs do not require auxiliary elements such as saturable absorbers. Unlike mode-locked lasers that generate short pulses, QCL combs emit nearly constant-intensity waveforms with a periodic, linear frequency modulation^9,10^ (Fig. 1e).

**Fig. 1:**
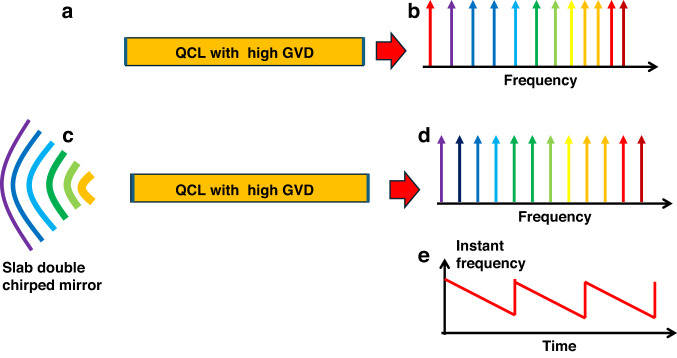
Impact of a double chirped grating refelctor onto QCL comb characteristics. **a** Quantum cascade laser (QCL) with a Fabry–Perot cavity formed by facet reflections. **b** Resonant mode frequencies in the Fabry–Perot cavity with group-velocity dispersion (GVD) present. **c** QCL with one facet replaced by an integrated double-chirped Bragg mirror. **d** Resonant mode frequencies with GVD compensated. **e** Instant frequency changes. linearly and periodically in time

Progress toward reliable, monolithic LWIR and THz combs, however, has been limited by group velocity dispersion (GVD)—the frequency dependence of the propagation velocity and propagation time of light in the gain medium. In a conventional Fabry–Pérot QCL cavity (Fig. 1a), GVD leads to non-equidistant longitudinal modes (Fig. 1b), which disrupts the phase locking necessary for comb operation. As a result, while such devices emit on multiple spectral lines, the phases are uncorrelated, limiting their utility for precision measurements.

To address this challenge, numerous GVD-compensation strategies have been investigated^11^, but most involve trade-offs between optical loss, dispersion control, and spectral bandwidth. In a recent study, Zeng et al. ^12^ introduced a novel approach employing a slab concave double-chirped Bragg mirror ^13^ (Fig. 1c) to replace the cleaved facets. This mirror comprises deep, periodically etched trenches in the semiconductor, with both the period and duty cycle linearly chirped. The resulting frequency-dependent reflection phases compensate for GVD-induced phase delays, effectively equalizing the round-trip time for photons across the emission spectrum. The combined chirp in period and amplitude significantly expands the design space, enabling fine control over reflection phase and precise GVD compensation. Consequently, the cavity supports an equidistant mode spectrum (Fig. 1d), facilitating robust comb formation. An additional benefit of this approach is the high refractive index contrast between semiconductor and air, which allows the compensating structure to remain compact and introduces minimal cavity loss.

Following detailed design, devices were fabricated using electron-beam lithography and dry etching. Comparative characterization of these dispersion-compensated QCLs and conventional Fabry–Pérot devices revealed that, although both exhibited emission bandwidths exceeding 3 THz, only the compensated devices showed a narrow RF beat note—a definitive hallmark of coherent frequency comb operation.

This work demonstrates, for the first time, stable LWIR frequency comb generation in a fully monolithic QCL operating at room temperature. These results represent a significant advance toward compact, chip-scale metrological and spectroscopic systems in the technologically important LWIR spectral range.
